# Early nail involvement in mycosis fungoides with rapid systemic progression: rethinking the role of Ki-67: a case report

**DOI:** 10.1186/s13256-025-05582-8

**Published:** 2025-10-31

**Authors:** Shafagh Ali Asgarzadeh, Amir Arshia Beheshti

**Affiliations:** 1 Department of Internal Medicine, School of Medicine, Ardabil University of Medical Sciences, Ardabil, Iran; 2 Students Research Committee, School of Medicine, Ardabil University of Medical Sciences, Ardabil, Iran

**Keywords:** Mycosis fungoides, Nail involvement, Ki-67, TOX, Cutaneous T-cell lymphoma

## Abstract

**Background:**

Mycosis fungoides, the most common cutaneous T-cell lymphoma, typically exhibits a gradual progression. However, aggressive forms with extracutaneous involvement and low proliferative indices may present diagnostic and prognostic challenges. Nail involvement appears to be infrequent and poorly understood, and is often considered a late-stage manifestation. The Ki-67 index, although frequently utilized as an indicator of tumor aggressiveness, may have limitations in specific mycosis fungoides subtypes that appear to be influenced by distinct molecular or immunological pathways.

**Case presentation:**

We present a 62-year-old Iranian woman with early stage mycosis fungoides, exhibiting erythematous abdominal plaques, pruritus, and early onset onychodystrophy. Within 4 months, the lesions rapidly progressed to painful plaques affecting the face and intertriginous areas. The initial biopsy revealed features consistent with plaque-stage mycosis fungoides, characterized by CD4 predominance, CD7 deficiency, and a low Ki-67 index (< 5%). Despite low proliferation indices, the disease progressed rapidly. Imaging revealed bilateral lymphadenopathy, and a subsequent biopsy confirmed ongoing disease, with limited CD8 expression, absent B-cell markers, and Ki-67 levels of 1–3%. The treatment regimen comprised methotrexate, interferon-alpha, and psoralen and ultraviolet A therapy. Partial cutaneous response was achieved; however, systemic progression occurred. Laboratory results revealed leukocytosis, elevated lactate dehydrogenase levels, and hepatic impairment. Bone marrow biopsies suggested early dissemination. Peripheral blood flow cytometry and nail biopsy were not conducted. The patient ultimately developed multiorgan failure and was transitioned to palliative care.

**Conclusion:**

This case suggests the potential for aggressive mycosis fungoides behavior despite indolent histopathological characteristics. Initial nail involvement might serve as a clinical marker of atypical progression. The limitations of Ki-67 alone suggest the need for comprehensive prognostic models that incorporate molecular biomarkers, such as thymocyte selection-associated high mobility group box, CD30, and T-cell receptor clonality.

## Introduction

Mycosis fungoides (MF) is the predominant subtype of cutaneous T-cell lymphoma (CTCL), generally progressing from patch to plaque and tumor stages in a gradual manner [1]. Some atypical variations, characterized by rapid diffusion and noncutaneous involvement, may present considerable diagnostic and prognostic challenges [2]. Nail involvement in MF appears to be exceptionally uncommon and underreported, often appearing as a late-stage incidental finding rather than an early clinical indicator [3]. The Ki-67 proliferation index, frequently utilized to evaluate tumor aggressiveness, may have limited reliability in certain MF subtypes, particularly those that appear to be influenced by alternative immunologic or epigenetic processes [4].

This case report describes an early stage mycosis fungoides with nail involvement and what appears to be a surprisingly rapid systemic progression, despite persistently low Ki-67 values. This particular clinical course suggests potential limitations in conventional prognostic assumptions and may indicate the need for additional biomarkers, including TOX, CD30, and T-cell receptor (TCR) clonality.

This paper aims to contribute insights into the early identification of aggressive MF phenotypes using a comprehensive clinical timeline, histopathologic correlation, and conceptual prognostic approach. This study suggests the potential significance of customized risk assessment by focusing on underrepresented manifestations in non-Caucasian populations and resource-constrained environments, possibly enhancing our understanding of MF’s biological heterogeneity.

## Case presentation

**Fig. 1:**
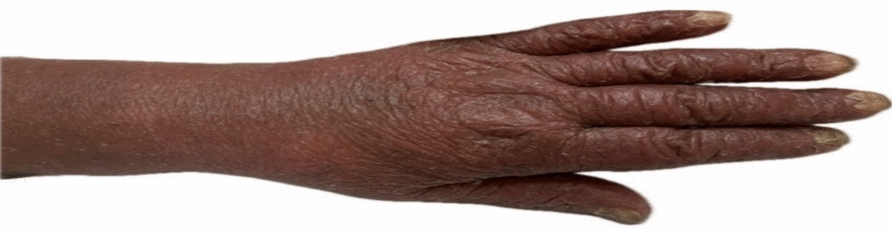
Early onychodystrophy in mycosis fungoides, possibly suggesting aggressive progression despite low Ki-67 index

This case report illustrates potential diagnostic and prognostic challenges that may be associated with mycosis fungoides (MF), particularly in cases involving what appears to be atypical nail involvement and rapid progression despite low Ki-67 proliferation indices. The patient, a 62-year-old Iranian woman with no previous history of cardiovascular, respiratory, viral, or metabolic diseases, presented in July 2024 with erythematous abdominal lesions and slight itching. The lesions initially exhibited mild scaling and atrophy, progressing over 4 weeks into hyperpigmented, indurated plaques accompanied by desquamation and onychodystrophy—a relatively uncommon manifestation in MF (Fig. 1).

**Fig. 2:**
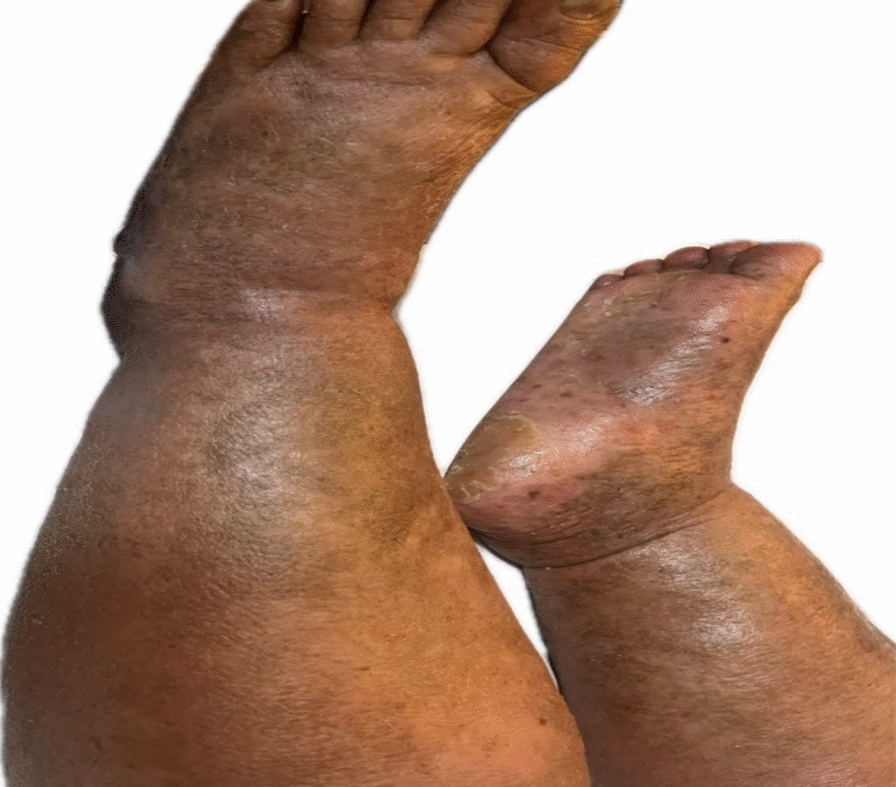
Lower limb edema and hyperpigmented plaques in advanced mycosis fungoides, suggestive of systemic progression

Upon examination, the patient exhibited lower limb edema (Fig. 2), suggesting possible systemic involvement consistent with the noted rapid clinical deterioration. She did not present with systemic B symptoms.

Skin involvement constituted approximately 15% of the body surface area (T2), according to International Society for Cutaneous Lymphomas/European Organisation for Research and Treatment of Cancer (ISCL/EORTC) criteria. A punch biopsy of an abdominal plaque revealed features consistent with plaque-stage mycosis fungoides: epidermotropism of atypical lymphocytes, Pautrier's microabscesses, and band-like lymphoid infiltration in the papillary dermis. Immunohistochemical staining was conducted using conventional techniques utilizing monoclonal antibodies targeting CD3, CD4, CD8, CD30, TOX, and Ki-67, all obtained from Dako, Denmark. Both positive and negative controls were incorporated.

A board-certified dermatopathologist conducted the histopathologic assessment. Immunohistochemistry (IHC) profile showed a predominance of CD4 + T cells, reduced CD7 expression, and a low Ki-67 index (< 5%). A nail biopsy was not conducted. Histopathological and immunophenotypic analyses were consistent with mycosis fungoides while helping to exclude differential diagnoses, including psoriasis, cutaneous sarcoidosis, and discoid lupus erythematosus. Immunohistochemical analysis supported the diagnosis and enabled timely commencement of treatment.

These findings suggested that this particular case of early-stage MF appeared to display exceptionally aggressive clinical behavior. By November 2024 (approximately 4 months following the biopsy), the patient experienced exacerbated pruritus, painful exudative plaques on the lower extremities, and newly formed lesions on the face, upper extremities, and intertriginous regions. CT imaging revealed bilateral inguinal lymphadenopathy, atherosclerotic changes, and a 10 mm umbilical hernia. A subsequent biopsy confirmed ongoing CD4 predominance, low CD8 expression, a Ki-67 index of 1–3%, and the absence of B-cell markers (CD20, CD79a, PAX5).

Despite the consistently low Ki-67 levels, the clinical course in this case was unexpectedly aggressive, suggesting a potential divergence between clinical and pathological features.

**Fig. 3:**
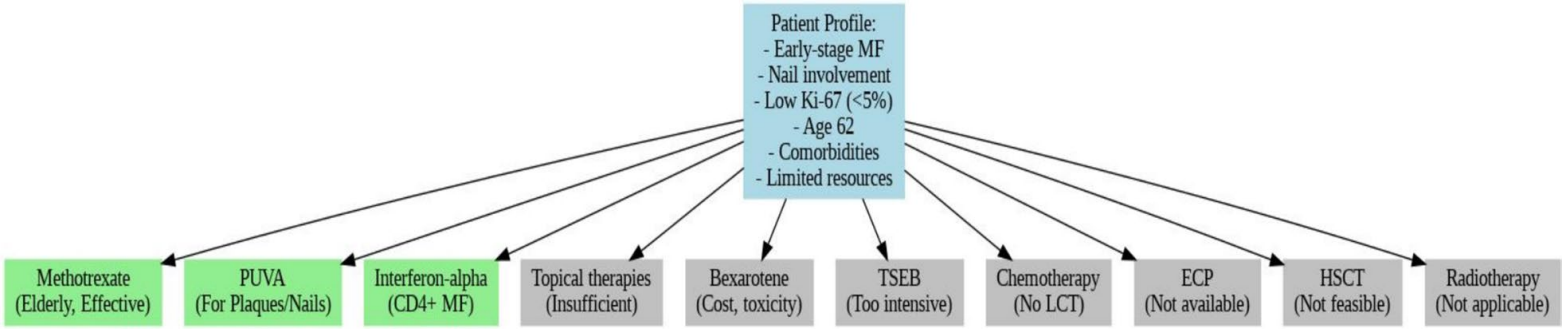
Treatment timeline and therapeutic interventions in mycosis fungoides: methotrexate, interferon-alpha, and PUVA therapy

Treatment was initiated in December 2024, consisting of methotrexate (15 mg/week), subcutaneous interferon-alpha (3 million IU, administered three times weekly), and PUVA (8-methoxypsoralen, 0.6 mg/kg, with UVA commencing at 1.0 J/cm^2^, administered biweekly). The selection of these treatments was based on the disease stage, lesion extent, and CD4-dominant immunophenotype. Corticosteroids, bexarotene, total skin electron beam (TSEB), extracorporeal photopheresis (ECP), or hematopoietic stem cell transplantation (HSCT) were not utilized due to clinical constraints or lack of availability (Fig. 3).

**Fig. 4:**
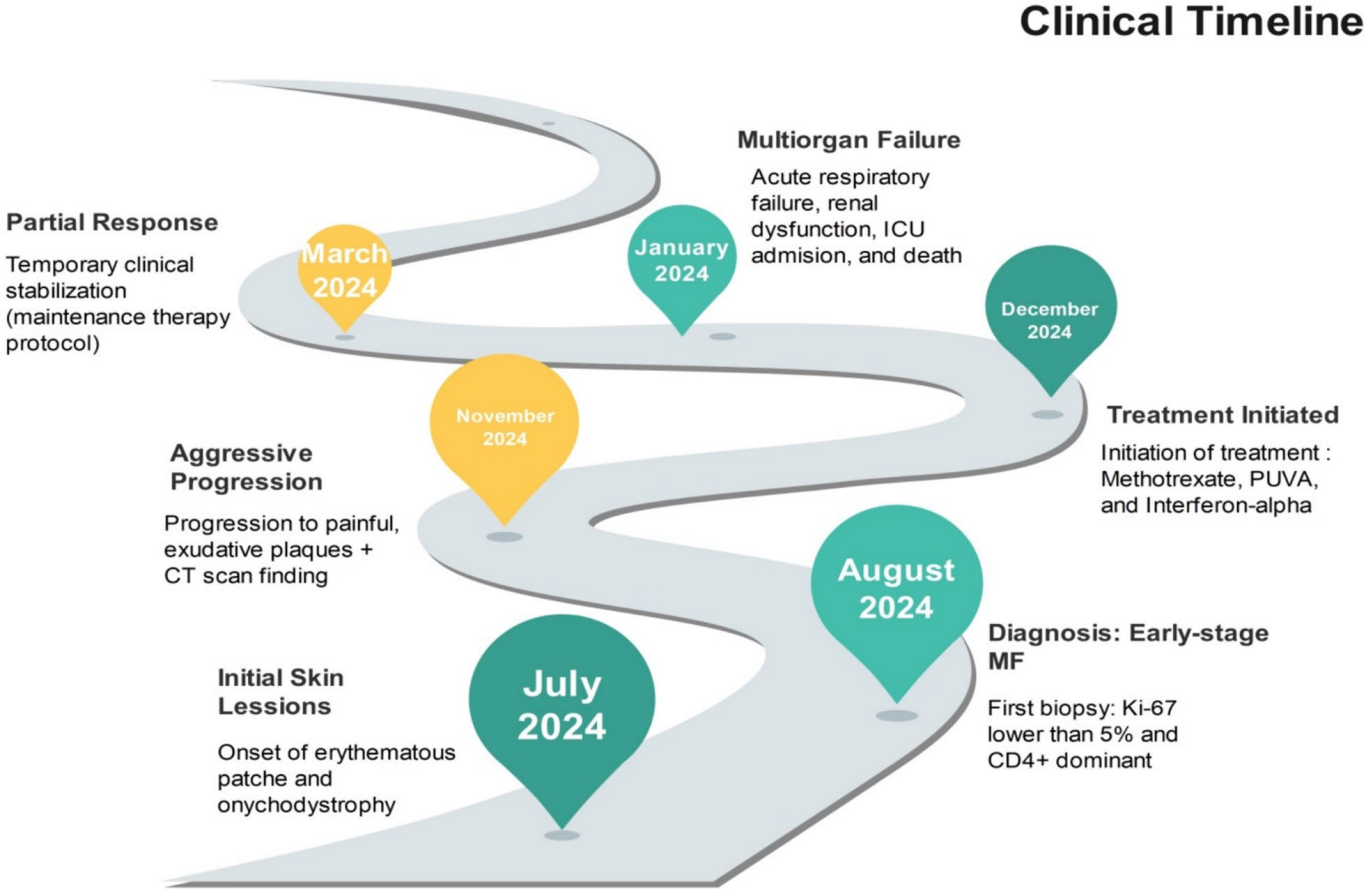
Clinical progression timeline showing rapid deterioration from early cutaneous lesions to multiorgan failure despite low Ki-67 index

Despite achieving partial cutaneous stabilization, systemic decline persisted. Laboratory results showed elevated LDH (1100 U/L), leukocytosis, increased C-reactive protein (CRP), and hepatic steatosis. The bone marrow biopsy suggested early hematologic dissemination. Peripheral blood flow cytometry was not conducted. The patient ultimately developed multiorgan dysfunction and respiratory failure in March 2025. Due to deteriorating renal and hepatic function, she was transitioned to palliative care. This case illustrates the potential for rapid progression in early stage MF and suggests possible limitations in the prognostic reliability of Ki-67 in certain clinical scenarios (Fig. 4).

## Discussion

**Fig. 5:**
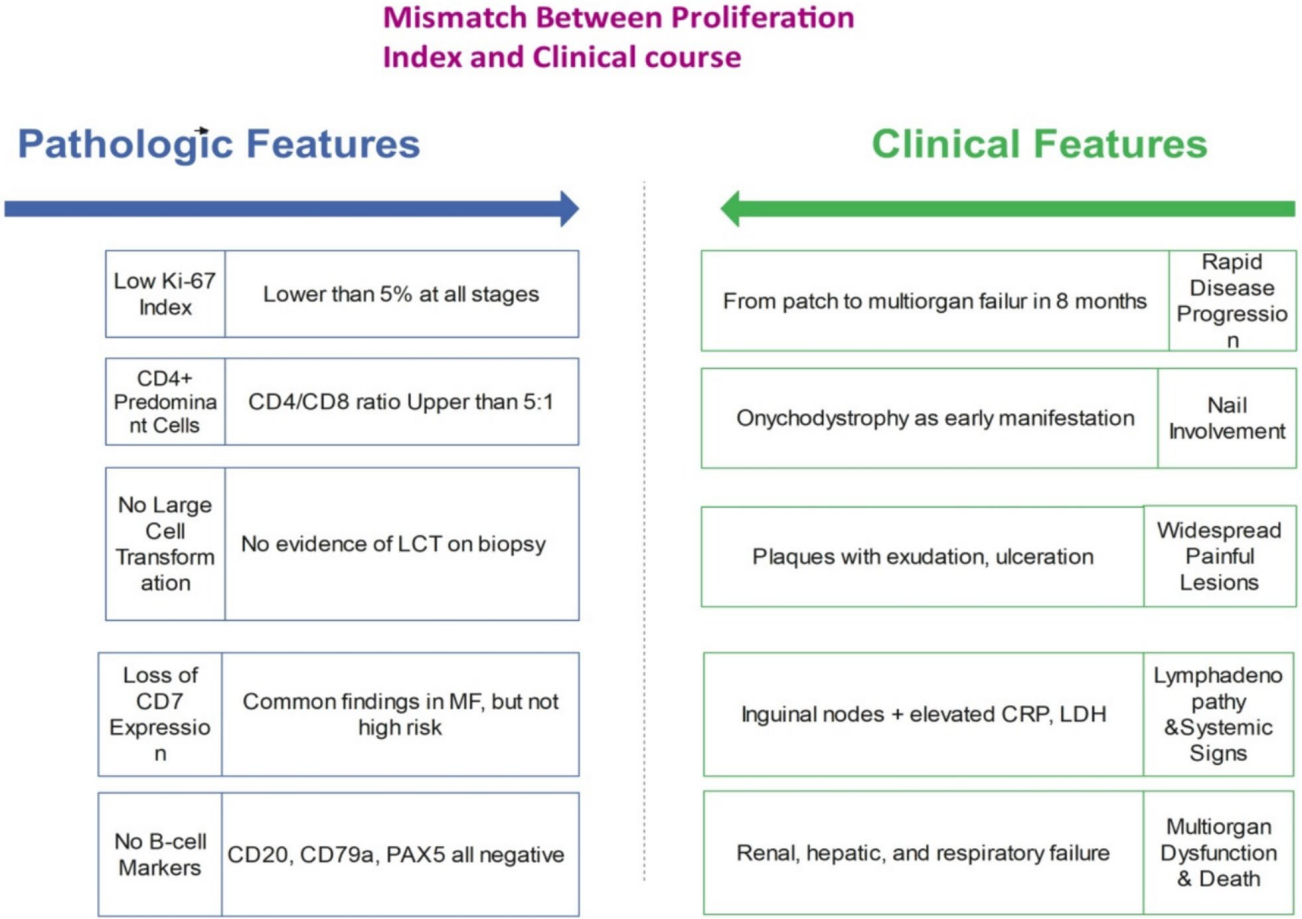
Apparent mismatch between pathologic features and clinical course in mycosis fungoides. Despite low-risk histopathology (e.g., low Ki-67, no LCT), this patient exhibited rapid systemic progression and multiorgan failure

This case illustrates a diagnostically complex manifestation of early stage mycosis fungoides (MF) in a Middle Eastern patient, characterized by relatively uncommon features such as initial nail involvement and what appeared to be unexpectedly rapid systemic progression, despite consistently low Ki-67 expression. Conventionally, Ki-67 is often considered a generally dependable indicator of cellular proliferation and tumor aggressiveness in MF [5]. However, in this particular case, the clinical course appeared to diverge from this typically anticipated pattern, as the patient progressed from restricted cutaneous involvement to widespread lymphadenopathy within months, suggesting potential limitations of Ki-67 as an isolated prognostic indicator in certain cases. Figure 5 depicts this apparent contradiction, with a low Ki-67 proliferation index (< 5%) despite systemic spread.

This discordance might suggest a possible broader spectrum of proliferation-discordant mycosis fungoides phenotypes, wherein oncogenic activity may advance through various molecular pathways. Further research in this area could be valuable, as it might lead to potential advancements in MF understanding. The transcription factor TOX (thymocyte selection-associated high mobility group box), which is often overexpressed in MF, may facilitate chromatin remodeling and repress differentiation-related genes, potentially promoting malignant persistence in a nonproliferative state [6, 7]. TOX has been associated with T-cell exhaustion, suggesting a possible connection between immune evasion and accelerated development [8].

Furthermore, the nail matrix might function as a potential refuge for malignant T cells, similar to central nervous system involvement in other lymphomas [9]. This location, being largely immune-privileged and subjected to minimal clinical examination, could potentially facilitate early clonal growth and contribute to treatment resistance [10].

The early manifestation of onychodystrophy in this particular case might suggest the initial dissemination of malignant clones rather than late-stage progression, raising questions about our understanding of nail involvement in MF [3]. One possible explanation pertains to T-cell receptor (TCR) clonality and CD30 expression, both of which were not accessible in our context. Aberrant TCR clonotypes, particularly those lacking functional diversity, have been associated with unfavorable outcomes in MF, even in cases with low Ki-67 levels [11]. Similarly, diminished or absent CD30 expression in rapidly progressing cases might indicate a departure from conventional cytotoxic pathways, potentially suggesting resistance to CD30-targeted therapies, such as brentuximab [12].

These observations may help elucidate the clinical–pathologic discrepancy reported in this case, suggesting that Ki-67 negativity may not exclude aggressive behavior and might benefit from assessment in conjunction with supplementary markers and clinical characteristics.

**Fig. 6:**
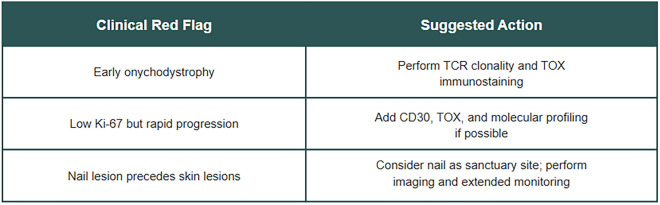
Proposed clinical alert system for atypical mycosis fungoides presentations, highlighting potential red flags and suggested diagnostic actions

In light of these preliminary insights, we suggest considering a clinical alert algorithm for atypical MF cases presenting with early nail involvement: (Fig. 6).

This approach suggests TOX and TCR analysis as potential frontline adjuncts in cases with apparent incongruent clinical and proliferative findings, particularly in resource-constrained settings where genomic assays are limited.

The apparent inconsistency between low Ki-67 levels and rapid development has been rarely documented. A few comparable cases of MF have been presented, characterized by low Ki-67 and rapid extracutaneous dissemination, suggesting that proliferation-independent mechanisms—such as immune evasion or microenvironmental modulation—might contribute to some aggressive MF characteristics [13]. Some researchers have questioned the universal applicability of Ki-67, noting that its expression may vary depending on the stage and subtype of the disease [4]. Despite this, such reports remain limited, especially among Middle Eastern populations. The underrepresentation of this population in CTCL research may be partially attributed to geographical factors, including delayed diagnosis, healthcare accessibility, and possible genetic or immunologic predispositions [14].

This scenario provides distinctive regional data that may question existing assumptions and suggests the potential need for more comprehensive predictive models. Nail involvement in MF appears to be inadequately described and is generally recorded primarily in case reports. These findings are consistent with our observations and support the hypothesis that nail abnormalities—especially when manifesting early—might signify systemic biological activity rather than solely local skin involvement.

**Fig. 7:**
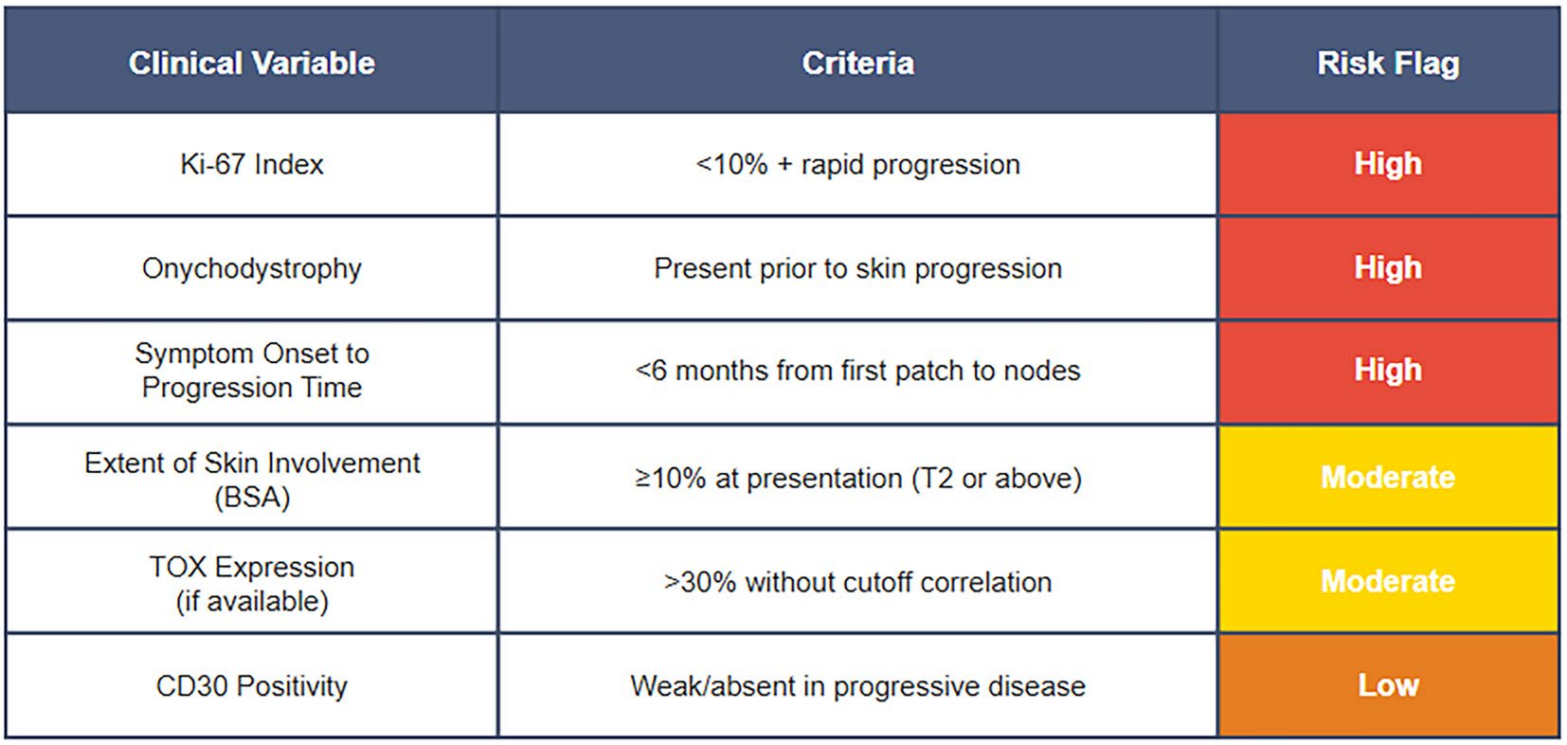
Proposed preliminary risk stratification tool for early stage mycosis fungoides based on clinical variables, criteria, and corresponding potential risk flags

On the basis of these preliminary observations, we suggest considering a Preliminary Prognostic Flagging (PPF) framework for early stage MF, particularly in resource-limited settings. This hypothetical model attempts to integrate standard and potentially relevant clinical indicators, including nail changes, that might help stratify patients at baseline. The goal would not be to replace molecular diagnostics but rather to potentially supplement them in settings where such tools may be unavailable (Fig. 7).

Interpretation:

- 2 or  more = consider early molecular testing, imaging, and systemic monitoring.
- 1  + 2  = closer surveillance and multidisciplinary review.
- Only  or  = monitor under standard guidelines.

The observations derived from this single patient case are inherently limited. The lack of a nail biopsy and molecular profiling significantly limits our understanding of the pathological and mechanistic aspects of the condition. This case may suggest considering a broader prognostic perspective in MF, particularly in resource-constrained environments. The proposed PPF framework serves as a preliminary, hypothesis-generating instrument that emphasizes readily observable but potentially underutilized variables.

Future research directions might include:

- Investigation of nail involvement as a potential prognostic indicator in larger, multi-institutional cohorts.
- Development of comprehensive prognostic models that integrate molecular, clinical, and microenvironmental factors.
- Regional data collection to address ethnic and structural health disparities in CTCL outcomes.

## Conclusion

This case illustrates a relatively uncommon manifestation of early stage mycosis fungoides, characterized by initial nail involvement and what appeared to be rapid systemic progression, despite low Ki-67 expression. The observed clinical–pathologic dissociation in this particular case suggests potential limitations of relying exclusively on proliferative indicators for prognostic evaluation. Alternative biomarkers, including TOX, CD30, and TCR clonality, might provide a more comprehensive understanding of disease aggressiveness in certain cases. In resource-constrained environments, early clinical manifestations—such as onychodystrophy—might warrant increased monitoring. This case suggests the potential value of integrated diagnostic models that combine clinical, histologic, and molecular indicators. Such models, with further validation, might contribute to enhanced risk stratification in atypical MF presentations.

## Methods

This work has been reported in line with the CARE criteria [15].

## Data Availability

Data and materials supporting the findings of this study are available from the corresponding author upon reasonable request.

## Abbreviations

MF: Mycosis fungoides
CTCL: Cutaneous T-cell lymphoma
PUVA: Psoralen and ultraviolet A therapy
TOX: Thymocyte selection-associated high mobility group box
TCR: T-cell receptor
LDH: Lactate dehydrogenase
CRP: C-reactive protein
IHC: Immunohistochemistry
C: Cluster of differentiation
HSCT: Hematopoietic stem cell transplantation
ECP: Extracorporeal photopheresis
TSEB: Total skin electron beam
ISCL: International Society for Cutaneous Lymphomas
EORTC: European Organisation for Research and Treatment of Cancer
